# Intercellular communication between peripheral monocytes and central nervous system cells in stroke

**DOI:** 10.4103/NRR.NRR-D-25-00738

**Published:** 2025-10-30

**Authors:** Masato Kanazawa, Masahiro Hatakeyama

**Affiliations:** 1 Faculty of Rehabilitation, Niigata University of Health and Welfare, Niigata, Japan; 2 Department of Neurology, Brain Research Institute, Niigata University, Niigata, Japan

**Keywords:** cell therapy, cell-cell communication, chemokines, cytokines, enteric microbiota, extracellular vesicles, macrophage, monocytes, mononuclear cell, peripheral blood mononuclear cells

## Abstract

Stroke, particularly ischemic stroke, induces dynamic interactions between peripheral monocytes and central nervous system cells, influencing neuroinflammation, repair, and functional recovery. Monocytes infiltrate the brain post-stroke and differentiate into macrophages, which interact with microglia, astrocytes, endothelial cells, and neurons. These interactions, mediated by chemokines, cytokines, and extracellular vesicles, can be detrimental or beneficial depending on context. Another interface is the gut–immune–brain axis, wherein gut microbiota, immune cells, and central nervous system-resident populations engage in reciprocal communication. Emerging therapies targeting monocyte subsets, their recruitment, and communication pathways. These include preconditioned peripheral blood mononuclear cells, bone marrow-derived mononuclear cells, mesenchymal stem cells, and cell-free or cell-mediated approaches utilizing the secretome. Together, these interventions hold promise for enhancing stroke recovery by modulating the immune–neural interface. This review summarizes recent advances in monocyte–central nervous system communication and its translational potential in stroke.

## Introduction

Stroke remains a leading cause of long-term disability and mortality worldwide (GBD 2021 Diseases and Injuries Collaborators, 2024). Despite advances in acute reperfusion therapies, such as intravenous thrombolysis and mechanical thrombectomy, many patients experience incomplete neurological recovery, largely due to secondary neuroinflammation and limited endogenous regeneration (Otsu et al., 2020; Denorme et al., 2022; Ng et al., 2022; Albers et al., 2024; Goyal et al., 2025). Recent findings highlight the crucial role of peripheral immune cells, especially monocytes, in modulating post-stroke injury and repair (Wattananit et al., 2016; Otsu et al., 2023).

Historically, an immune-privileged organ, the central nervous system (CNS), is now known to actively interact with peripheral immune cells, particularly monocytes and other subsets of peripheral blood mononuclear cells (PBMCs) (Castellani et al.,2023). Stroke-induced blood-brain barrier (BBB) disruption facilitates the infiltration of PBMCs into the brain parenchyma, where they engage in complex bidirectional communication with resident microglia, astrocytes, neurons, and endothelial cells. These interactions can be either detrimental or beneficial, depending on the timing, cellular phenotype, and local microenvironment (Hatakeyama et al., 2020a; Park et al., 2022).

This review aimed to provide a comprehensive update on intercellular communication between peripheral monocytes and CNS-resident cells following stroke. We explored the dynamics of monocyte subsets and the mechanisms mediating PBMC–CNS cross-talk (Wattananit et al., 2016; Park et al., 2022; Otsu et al., 2023), including protein–protein interactions, extracellular vesicles (EVs), and non-coding RNAs (Wang et al., 2020; Angerfors et al., 2023; Choi et al., 2023), as well as emerging therapeutic strategies aimed at modulating these interactions. By delineating the immunological underpinnings of stroke pathology and recovery, we aimed to highlight novel translational approaches in harnessing peripheral immune responses for therapeutic benefits.

## Search Strategy

A literature review was performed using PubMed in June and July 2025. We searched articles published between January 2010 and July 2025 using the following search terms: “stroke,” “cerebral ischemia,” “thrombolysis,” “mechanical thrombectomy,” and “cell therapy.” For the molecular aspect, we searched articles published between January 2000 and July 2025 using the following search terms: “stroke,” “cerebral ischemia,” “cerebral hemorrhage,” “monocytes,” “macrophages,” “peripheral blood mononuclear cells,” “chemokines,” “cytokines,” “extracellular vesicles,” and “microRNAs (miRNAs).” We also searched for such clinical trials in the National Institutes of Health Clinical Trial Database (ClinicalTrials.gov) using the following search terms: “stroke” and “stem cell.” Based on these data, except for neural stem cells, we selected completed clinical trials or ongoing studies using cell therapy for stroke (**Tables 1** and **2**).

**Table 1:** Selected completed clinical trials using cell therapy for stroke

ClinicalTrials.gov Identifier	Source	Administration timing after onset	Result
NCT03384433	Allogenic mesenchymal stem cell derived exosome miR-124 (Dehghani et al., 2022)	< 24 h	Safety
NCT02178657	Autologous, adult bone marrow-derived mononuclear cells (IBIS) (Moniche et al., 2023)	4–7 d	No statistically significant changes in the modified Rankin Scale score at 180 d
		(median 5 d)	
NCT02961504	Allogeneic, adult bone marrow-derived progenitor stem cells (MultiStem^®^, TREASURE) (Houkin et al., 2024	18–36 h	No statistically significant changes in the modified Rankin Scale score at 90 d
	Allogeneic, adult bone marrow-derived mesenchymal stem cells (Muse cells) CD105(+)	14–28 d	Muse cell group exhibited statistically significant improvements in upper limb function scales.
	SSEA-3(+) (Niizuma et al., 2023)		
NCT01287936	Allogeneic, adult bone marrow-derived mesenchymal stem cells.	6–60 mon	No statistically significant changes in the modified Rankin Scale score in 2 yr
	(SB623)		
	CD29(+) CD90(+) CD105(+) CD34(–)		
	CD45(–) (Steinberg et al., 2018)		
NCT01297413	Allogenic mesenchymal stem cell under low oxygen (5%) conditions (Levy et al., 2019)	> 6 mon	Statistically significant changes in the Berthal Index scores at 12 mon

## Peripheral Monocytes and Peripheral Blood Mononuclear Cell Dynamics in Stroke

### Recruitment and infiltration

Following ischemic stroke or intracerebral hemorrhage, a systemic inflammatory cascade is rapidly initiated, resulting in the mobilization of monocytes from the bone marrow and spleen into circulation. These cells are actively recruited to the brain via a chemokine gradient. Notably, the chemokines C-X-C motif chemokine ligand 12 (CXCL12) (also known as stromal cell-derived factor 1 [SDF-1]) (Hill et al., 2004) and C-C motif chemokine ligand 2 (CCL2) (also known as monocyte chemoattractant protein-1) (Fang et al., 2018) are upregulated in response to CNS injury. Monocytes expressing C-X-C chemokine receptor type 4 (CXCR4) and C-C motif chemokine receptor 2 (CCR2) migrate along CXCL12 and CCL2 gradients, respectively. These monocytes penetrate the compromised BBB and accumulate in the perivascular space and the infarct core (Kanazawa et al., 2015).

In rodent models of transient middle cerebral artery occlusion, Ly6C^hi^ monocytes infiltrate the ischemic brain as early as 6–12 hours post-occlusion, peaking after approximately 3 days (Kanazawa et al., 2015; Fang et al., 2018). Interestingly, in an ischemic stroke model, CXCL12 signaling has been reported to promote the mobilization of neural progenitor cells to the ischemic lesion and promote tissue repair (Robin et al., 2006). Similarly, in intracerebral hemorrhage, PBMC infiltration correlates with hematoma size and secondary injury progression due to inflammation (Jiang et al., 2020). This early influx is largely composed of classical pro-inflammatory monocytes, which contribute to both debris clearance and secondary tissue injury through the release of cytokines, such as tumor necrosis factor-alpha (TNF-α), interleukin-1beta (IL-1β), and matrix metalloproteinase-9. Conversely, the later recruitment of Ly6C^lo^ non-classical monocytes is associated with anti-inflammatory responses and tissue remodeling.

### Temporal and functional subsets post-stroke

**Figure 1:**
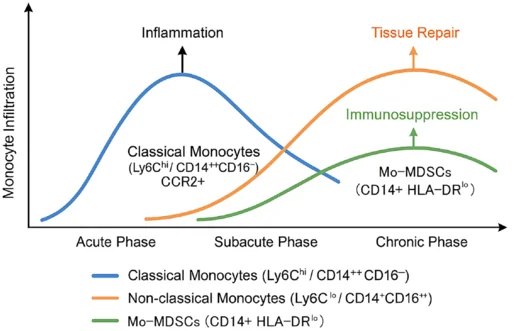
Dynamic changes of the monocyte subsets after ischemic stroke. CD: Cluster of differentiation; HLA: human leukocyte antigen; Mo-MDSCs: monocytic myeloid-derived suppressor cells.

Monocyte subset behavior evolves across the acute (minutes–days), subacute (days–weeks), and chronic (a week or later) phases of stroke (**Figure 1**; Planas, 2018). Broadly, stroke triggers a systemic inflammatory response that mobilizes CCR2^+^ classical monocytes from the bone marrow and spleen into the blood, where they flood into the injured brain. In contrast, Ly6C^lo^ patrolling monocytes entered later, often in smaller numbers. Furthermore, monocytic myeloid-derived suppressor cells, characterized by human leukocyte antigen (HLA)-DRlo cluster of differentiation (CD)14+ markers and arginase-1 (Arg1) expression, have emerged as key regulators of post-stroke immunosuppression (Achmus et al., 2020). These cells are enriched in the circulation of patients during the early subacute stroke phase, modulating T cell activity and peripheral tolerance.

Human monocytes can be classified into three major subsets based on CD14 and CD16 expression: classical (CD14^++^CD16^−^), intermediate (CD14^++^CD16^+^), and non-classical (CD14^+^CD16^++^) (Thomas et al., 2015, 2017; Bai et al., 2023). Analogous populations in mice are defined by Ly6C and chemokine receptor profiles (Ly6C^hi^/CCR2+ *versus* Ly6C^lo^/CX3CR1^hi^). Each subset exhibits distinct migratory behaviors, cytokine production profiles, and interaction patterns with resident CNS cells. (1) Classical monocytes dominate the acute phase and exhibit pro-inflammatory phenotypes via CCR2/CCL2 signaling in the acute phase (Days 1–3). These release pro-inflammatory cytokines, such as IL-1β and TNF-α, exacerbating neuroinflammation. These factors contribute to debris clearance and vascular stabilization in the ischemic core. However, excessive infiltration can disrupt the BBB. Non-classical Ly6C^lo^ monocytes remain relatively quiescent in the very acute phase. Experimental depletion of Ly6C^lo^ cells did not change the early infarct size or immune activation (Michaud et al., 2014). (2) Non-classical monocytes are involved in patrolling, promoting inflammation resolution, and reparative processes via the C-X3-C motif ligand 1(CX3CL1)/ C-X3-C motif) receptor 1 (CX3CR1) axis during the subacute (Days 3–7) and chronic phases (Tang et al., 2014). A shift toward Ly6C^lo^/CCR2- monocytes promotes tissue remodeling and suppresses inflammation. The monocytes secrete anti-inflammatory cytokines, such as transforming growth factor-beta (TGF-β) and IL-10, neutralizing pro-inflammatory signals and promoting astrocytic glial scar formation. (3) Intermediate monocytes may act as transitional forms with mixed properties.

Monocyte levels in the blood and brain gradually normalize, while macrophages persist in the tissues after stroke. The persistent monocyte-derived macrophages (MDMs) interact with microglia to influence recovery (Wattananit et al., 2016). MDMs producing neurotrophic factors, such as TGF-β and insulin-like growth factor 1, supporting axonal regeneration (Wattananit et al., 2016; Ge et al., 2017). In response to stroke, repair-associated microglia emerge in the damaged brain during the phase of angiogenesis and cerebrovascular remodeling (Mastorakos et al., 2021). These cells express vascular endothelial growth factor A (VEGF-A) (Kanazawa et al., 2017a; Otsu et al., 2023), a potent proangiogenic factor, and are juxtaposed to areas of injured cerebrovasculature by communicating with each other. The selective depletion of Ly6C^hi^ monocytes and inflammatory monocyte/macrophage transfer do not impact neural recovery after photothrombotic stroke (Schmidt et al., 2017). Although the differences in experimental models (middle cerebral artery occlusion versus photothrombus) might be impacted, their role in tissue repair remains unclear.

Additionally, monocytic myeloid-derived suppressor cells, characterized by CD14^+^HLA-DR^lo^ markers and Arg1 expression, have emerged as key regulators of post-stroke immunosuppression (Achmus et al., 2020). These cells are enriched in the patient’s circulation during the early subacute phase of stroke, particularly on days 3 and 5 post-stroke, and may modulate T cell activity and peripheral tolerance. Human data are limited and the increased number of monocytic myeloid-derived suppressor cells on day 5 was in a few patients. Therefore, further clinical sample analysis is needed.

Single-cell RNA sequencing has revealed dynamic changes in gene expression within both PBMCs and CNS cells during stroke, suggesting bi-directional reprogramming of cellular states (Cho et al., 2022; Zheng et al., 2022). The T cells exert divergent effects. Th1 and Th17 responses exacerbate the injury, while regulatory T cells (Tregs) may promote repair by inhibiting astrogliosis (Liesz et al., 2009; Ito et al., 2019). After a stroke, monocytes/macrophages secrete CXCL10 and recruit Th1 cells (Mantovani et al., 2004). Interferon-gamma derived from Th1 cells recruits CD4^+^ and CD8^+^ T cells (Yilmaz et al., 2006). A recent study showed that CXCR3/CXCL10-mediated brain infiltration and CD8^+^ T cells produced anti-inflammatory cytokines, such as epidermal growth factor-like transforming growth factor and IL-10 in CD8^+^ T cells (Cai et al., 2022). Post-stroke intravenous transfer of CD8^+^ T regulatory-like cells reduced infarct lesion, promoting long-term neurological recovery in young males or aged mice of both sexes. Thus, these unique CD8^+^ T cells serve as early responders to rally defense against stroke injuries to the brain, offering fresh perspectives for clinical translation.

## Mechanisms of Peripheral Blood Mononuclear Cell–Central Nervous System Cell Communication

PBMCs, including monocytes, T cells, and dendritic cells, influence stroke outcomes through multiple modes of interaction with CNS cells. These mechanisms include chemokine-receptor signaling, cytokine secretion, direct cell–cell contact, and the exchange of EVs containing regulatory RNA species. These interactions evolve over time and can exacerbate tissue injury and promote recovery.

### Protein-mediated signaling pathways

#### Chemokine–receptor axes

**Figure 2:**
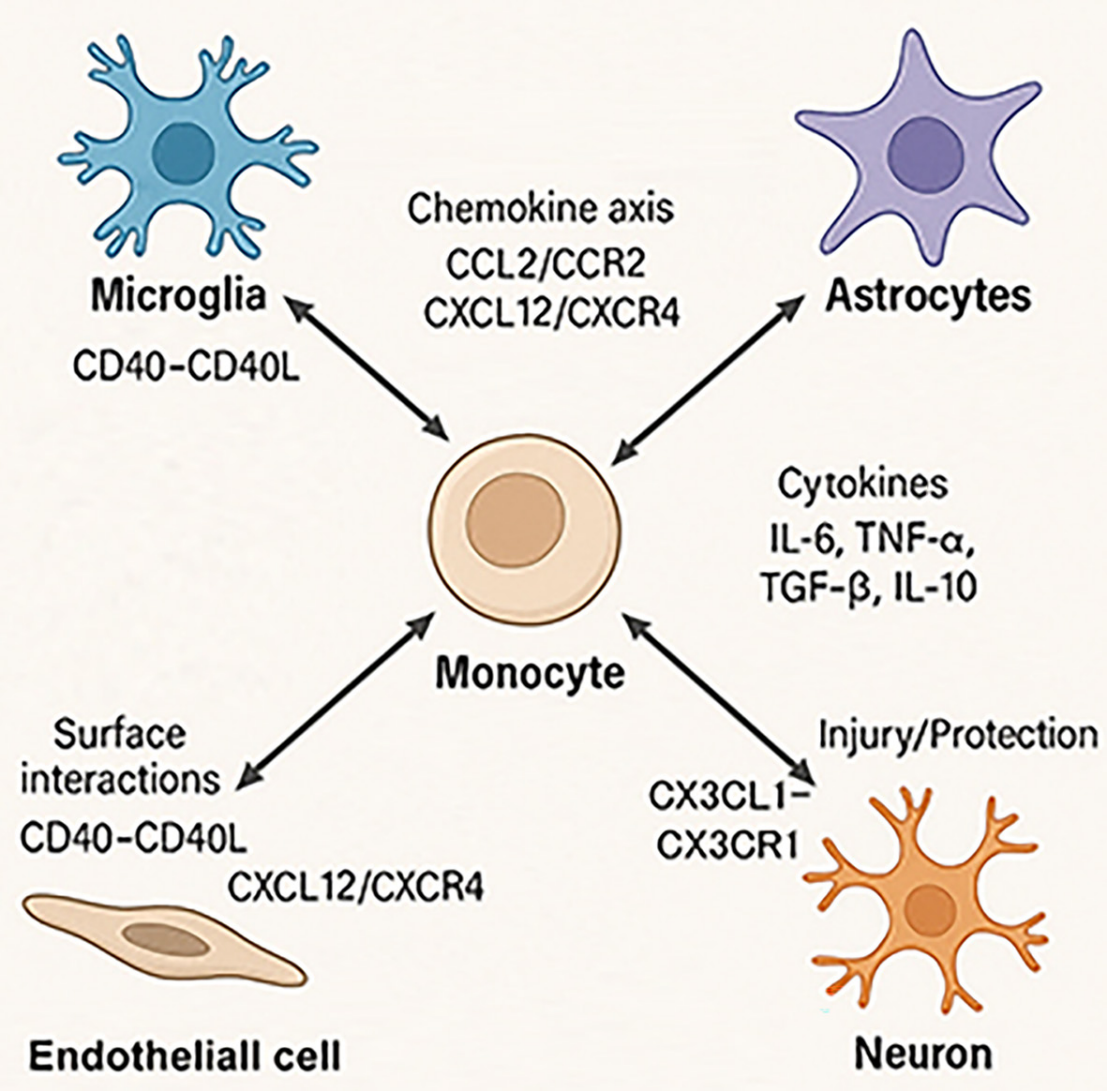
Monocytes and CNS cell interactions after ischemic stroke. Monocytes/macrophages communicate with CNS cells after an ischemic stroke. The C-C motif chemokine ligand 2 (CCL2)/C-C motif chemokine receptor 2 (CCR2) and C-X-C motif chemokine ligand 12 (CXCL12)/C-X-C chemokine receptor type 4 (CXCR4) axes are primarily connected. After communication, monocytes/macrophages produce inflammatory cytokines, such as IL-6 and TNF-α or anti-inflammatory cytokines such as TGF-β and IL-10. CD: Cluster of differentiation; CNS: central nervous system; CX3CL1: C-X3-C motif ligand 1; CX3CR1: C-X3-C motif receptor 1; HLA: human leukocyte antigen; IL: interleukin; Mo-MDSCs: monocytic myeloid-derived suppressor cells; TGF-β: transforming growth factor-beta; TNF-α: tumor necrosis factor-alpha.

Chemokine signaling is central to the recruitment and functional polarization of PBMCs in the post-stroke brain **(Figure 2)**: (1) CXCL12–CXCR4 axis: Upregulated in the ischemic brain, particularly in astrocytes and endothelial cells, while CXCL12 attracts CXCR4^+^ monocytes and neural progenitor cells to areas of injury (Hill et al., 2004; Robin et al., 2006; Kanayama et al., 2025). Experimental models have shown that this pathway enhances both neuroinflammation and vascular remodeling, depending on the timing and context. (2) CCL2–CCR2 axis: CCL2, primarily secreted by astrocytes and pericytes, is essential for the recruitment of classical CCR2+ monocytes during the acute phase of stroke (Che et al., 2001; Duan et al., 2018). These cells infiltrate the parenchyma, where they initially contribute to inflammation but later participate in debris clearance, angiogenesis, and extracellular matrix remodeling. Disruption of this axis (e.g., in CCR2-deficient mice) impairs recovery, indicating a dual role (Fang et al., 2018). Ly6C^hi^ monocytes upregulate TGF-β and VEGF by day 7, supporting neovascularization. Conversely, the failure of this switch (as in CCR2^–/–^ mice) leads to chronic deficits. (3) CX3CL1–CX3CR1 axis: Fractalkine (CX3CL1), produced by neurons, binds to CX3CR1 on microglia and monocytes, modulating their activation states (Tang et al., 2014). This axis appears to fine-tune inflammatory responses and support neuroprotective interactions under certain conditions.

#### Cytokine signaling

PBMCs secrete a diverse range of cytokines that influence resident CNS cells: (1) Pro-inflammatory cytokines, such as TNF-α, IL-1β, and interferon-gamma, amplify microglial activation, disrupt the BBB, and contribute to neuronal injury (Kanazawa et al., 2017a, b; Hatakeyama et al., 2019). (2) Anti-inflammatory cytokines, such as IL-10 and TGF-β, promote tissue repair, regulate astrogliosis, and support synaptic remodeling. These are typically produced by Ly6C^lo^ monocytes or Tregs in later phases (Liesz et al., 2009; Fang et al., 2018; Hatakeyama et al., 2019).

#### Costimulatory and adhesion molecules

CD40–CD40L interactions between activated T cells and microglia potentiate pro-inflammatory gene expression and promote leukocyte adhesion and migration across the BBB (Omari et al., 2004; Ishikawa et al., 2005).

Integrins such as very late antigen-4 (VLA-4) are upregulated on infiltrating T cells and mediate adhesion to CNS endothelial cells, facilitating parenchymal entry (Liesz et al., 2011). However, clinical trials targeting VLA-4 have not demonstrated efficacy in acute stroke (Elkind et al., 2020), underscoring the complexity of immune modulation.

### Therapeutic extracellular vesicles as mRNA carriers and cytokines

Following ischemic stroke, various brain-resident cells, including neurons, astrocytes, microglia, and endothelial cells, as well as peripheral immune cells, release EVs, including exosomes. These EVs are critical mediators of communication between PBMCs and CNS cells, and carry regulatory molecules, such as miRNAs and proteins, that can affect the behavior and function of recipient cells.

The selective packaging of miRNAs into EVs is regulated through RNA-binding proteins, such as hnRNPA2B1 and YBX1, which help determine cargo composition (Villarroya–Beltri et al., 2013; Shurtleff et al., 2016). After a stroke, EVs are often enriched with specific miRNAs, including miR-133b, miR-21-5p, miR-146a-5p, and the miR-17-92 cluster, which are implicated in neural repair and immune modulation (reviewed by Qiu et al., 2018 and Zhang et al., 2019). Additionally, other miRNAs have also attracted attention for their roles in post-stroke recovery.

#### miRNAs from peripheral blood mononuclear cells to central nervous system cells

miR-124 is delivered by microglial- or monocyte-derived EVs. miR-124 suppresses inflammatory gene expression and enhances neurogenesis and synaptic plasticity. It is one of the most consistent neuroprotective miRNAs identified in stroke models (Dehghani et al., 2022; Song et al., 2023). Intracerebral implantation of mesenchymal stem cell derived miR-124 showed no post-interventional adverse effects in five ischemic stroke patients (**Table 1**, registration number NCT03384433) (Dehghani et al., 2022).

miR-155-5p promotes microglial activation and cytokine release in pro-inflammatory PBMC-derived exosomes. Its inhibition reduces inflammation, prompts angiogenesis via VEGF signaling, and improves neurological outcomes after ischemic stroke (Caballero-Garrido et al., 2015; Otsu et al., 2023).

### Therapeutic extracellular vesicles as cytokine and mRNA carriers

Hypoxia-preconditioned microglia release EVs enriched in TGF-β. These EVs enhanced neuroprotective and anti-inflammatory effects in stroke models (Zhang et al., 2021).

Bone marrow-derived macrophages potentially enhance microglial phagocytosis via EVs following intracerebral hemorrhage. EVs-carried Arg1 from bone marrow-derived macrophages were found to augment microglial phagocytosis and hematoma clearance (Hu et al., 2025).

Engineered EVs targeting CXCR4^+^ monocytes represent novel delivery platforms for cancer treatment. Engineered EVs efficiently and specifically deliver either a bifunctional apoptotic peptide targeting CXCR4 to suppress leukemia or an antisense oligonucleotide to knockdown oncogenic miR-125b in epidermal growth factor receptor-expressing cancer cells, resulting in decreased cell viability (Jayasinghe et al., 2022).

## Crosstalk between Gut and Brain: The Gut–Immune–Brain Axis

Accumulating evidence shows that stroke triggers systemic responses that extend far beyond the brain. One critical interface is the gut–immune–brain axis, wherein gut microbiota, immune cells, and CNS-resident populations engage in reciprocal communication (Zhang et al., 2024).

**Figure 3:**
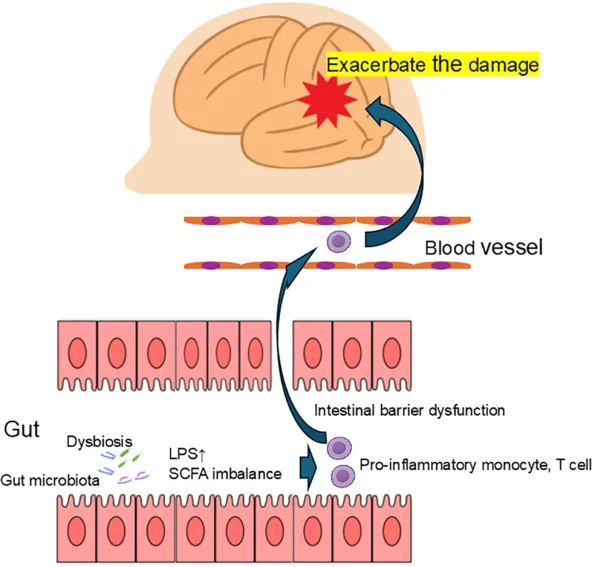
Systemic crosstalk between the gut microbiota, monocytes, and the brain after ischemic stroke. Gut microbiota dysbiosis, characterized by an imbalance in bacterial subpopulations, is a common consequence of stroke. This dysbiosis is marked by a reduction in beneficial metabolites such as short-chain fatty acids (SCFAs) and an increase in lipopolysaccharide (LPS) derived from gram-negative bacteria. Following ischemic stroke, these changes trigger a systemic inflammatory response, leading to elevated levels of pro-inflammatory cytokines, including tumor necrosis factor-α, interleukin (IL)-1, IL-6, and interferon-gamma. These cytokines prime circulating monocytes toward a pro-inflammatory phenotype, thereby enhancing their infiltration into the central nervous system and exacerbating ischemic brain injury.

After ischemic stroke, alterations in intestinal microbiota composition, termed gut dysbiosis, can exacerbate neuroinflammation. Stroke induces increased intestinal permeability and translocation of microbial components such as lipopolysaccharide and short-chain fatty acid imbalances, which activate Toll-like receptor signaling in immune cells (**Figure 3**; Xu et al., 2021). Monocytes exposed to these circulating danger signals undergo phenotypic changes, often adopting a more pro-inflammatory profile before infiltrating the CNS. Studies in rodent models have shown that depletion or manipulation of the gut microbiota significantly alters post-stroke immune responses. For example, germ-free mice or those treated with broad-spectrum antibiotics show attenuated monocyte infiltration and reduced infarct volumes (Winek et al., 2016; Benakis et al., 2020). Interestingly, transplanting fecal microbiota from stroke mice fed in a conventional environment worsens outcomes, while increased pro-inflammatory T cell differentiation and lymphocyte metastasis from the gut to the brain were observed in germ-free stroke mice (Singh et al., 2016). Interventions targeting the gut microbiota—such as probiotics (Liu et al., 2020), prebiotics (Yuan et al., 2021), short-chain fatty acid supplementation (Sadler et al., 2020), or fecal microbiota transplantation (Singh et al., 2016)—could serve as adjunctive strategies to modulate monocyte function. Modulating the gut–immune–brain axis may offer a route to transform the post-stroke immune landscape from pro-inflammatory to reparative, enhancing the efficacy of cell-based therapies.

## Therapeutic Strategies Targeting the Monocyte–Central Nervous System Axis

**Figure 4:**
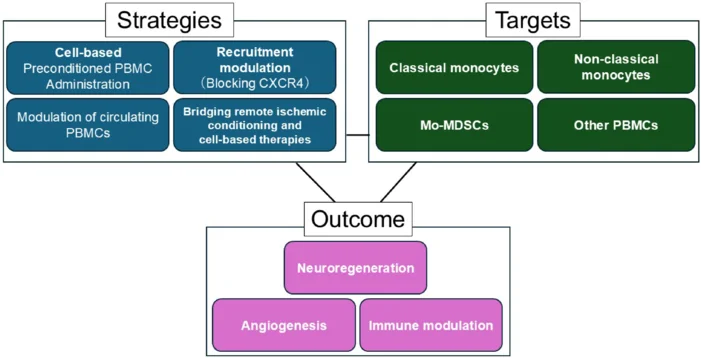
Monocyte-mediate stroke therapies. CXCR4: C–X–C chemokine receptor type 4; Mo-MDSCs: monocytic myeloid-derived suppressor cells; PBMCs: peripheral blood mononuclear cells.

Targeting peripheral monocytes and their interactions with CNS-resident cells offers a promising avenue for promoting neurorepair and limiting secondary injury after stroke. Emerging strategies range from cell therapies using autologous bone marrow-derived mononuclear cells (BM-MNCs) and PBMCs to the modulation of chemokine axes, immune reprogramming, and synergistic combinations such as rehabilitation and remote ischemic conditioning (RIC). Below, we review recent advances in therapeutic approaches aimed at harnessing or manipulating PBMC–CNS communication. However, it is unknown whether each strategy targets a specific cellular subtype, such as monocytes or PBMCs. Thus, it is necessary to determine their therapeutic effects (**Figure 4**).

### Cell therapy with autologous bone marrow mononuclear cells

BM-MNCs are a heterogeneous population that includes monocytes, hematopoietic stem/progenitor cells (e.g., CD34⁺ cells), lymphocytes, and mesenchymal stromal cells. Researchers identify and quantify the monocyte-containing fraction within BM-MNCs using flow cytometry and surface markers. Several ongoing and pending unpublished cell–based clinical trials now exist. We searched for such clinical trials in ClinicalTrials.gov. Based on these data, except for neural stem cells, we selected ongoing trials (**Table 2**). Moreover, selected major completed clinical trials are added in **Table 1** (Hatakeyama et al., 2020b; Dehghani et al., 2022; Kanazawa et al., 2024; Rust et al., 2024; Tang et al., 2024).

**Table 2:** Recent ongoing clinical trials using cell therapy for stroke

ClinicalTrials.gov Identifier	Source	Administration timing after onset
NCT04093336	Umbilical cord-derived mesenchymal stem cells	< 7 d
NCT04013646	Umbilical cord-derived mesenchymal stem cells with erythropoietin	From 30 d to less than 9 mon
NCT04953663	Allogeneic bone marrow-derived mesenchymal stem cells	More than 6 mon
NCT04590118	Allogeneic mesenchymal stem cells	More than 6 mon
NCT03545607	Allogeneic, adult bone marrow-derived progenitor stem cells (MultiStem^®^, MASTERS-2)	18–36 h
NCT02795052	Autologous bone marrow-derived stem cell	> 6 mon
NCT06752720	Autologous mesenchymal stem cell	Between 6 mon and 5 yr ago
NCT03570450	Adipose derived stem cells	6 mon after
NCT06138210	Exosomes derived from human induced pluripotent stem cells	Between 1 d and 7 d

Data are sourced from Kanazawa et al. (2024a).

A pilot clinical trial by Moniche et al. (2012) evaluated the safety and feasibility of intra-arterial transplantation of autologous BM-MNCs in patients with middle cerebral artery stroke. Approximately 2% of the transplanted cells were CD34^+^ hematopoietic stem cells, and monocytes were also present in the cell population. The study reported no serious adverse events and noted increased levels of β-nerve growth factor after transplantation, suggesting a potential neuroprotective effect. Subsequently, a meta-analysis of nine studies, including five randomized controlled trials, assessed the efficacy of BM-MNC therapy in stroke patients. While no significant improvements in neurological function were observed at 3 or 6 months post-transplantation, the treatment was consistently shown to be safe (Chumnanvej et al., 2020). These findings underscore the need for further trials to clarify its therapeutic value. Similarly, the phase 2 IBIS trial (Intra-arterial Bone marrow mononuclear cell Injection in Stroke) conducted in Spain did not demonstrate significant improvements in functional outcomes, as measured by the modified Rankin Scale at 180 days (Moniche et al., 2023). However, another group showed that the administration of autologous CD34^+^ mononuclear cells from bone marrow has shown promise in subacute stroke patients, with improvements in motor function and perfusion metrics (Taguchi et al., 2015).

Thus, allogenic adult bone marrow-derived mesenchymal stem cells such as MultiStem®, multi-lineage differentiating stress enduring (Muse) cells, and SB623 cells have shown statistically significant improvements in selected outcomes (Steinberg et al., 2018; Niizuma et al., 2023; Houkin et al., 2024). However, clinical efficacy remains to be proven in larger, controlled trials. It is also the first human stroke study to evaluate allogenic adult bone marrow-derived mesenchymal stem cells grown under hypoxic conditions, which favorably affects cell proliferation, gene expression, cytokine production, and migration (Levy et al., 2019). Administration of allogeneic ischemia-tolerant mesenchymal stem cells in patients with chronic stroke induced better outcome at 12 months (Levy et al., 2019). Further clinical trials are warranted to determine whether different time windows, patient populations, or cell preparations may enhance therapeutic outcomes (Tang et al., 2024).

### Cell therapy with autologous peripheral blood mononuclear cells

Preclinical studies have shown that autologous PBMCs, particularly when preconditioned with hypoxia or oxygen-glucose deprivation, can home to ischemic regions and contribute to tissue repair. These cells secrete a range of neurotrophic and angiogenic factors, including VEGF and TGF-β, which support neovascularization and neuronal survival (Hatakeyama et al., 2019; Otsu et al., 2023). Interestingly, we observed a significant reduction in the secretion of miR-155-5p, a proinflammatory miRNA, in oxygen-glucose deprivation-preconditioned PBMCs. This reduction sustains hypoxia-inducible factor-1α (HIF-1α) and VEGF signaling, thereby promoting regenerative responses in the host brain and supporting a novel concept we call “asymmetric miRNA regulation” (Otsu et al., 2023). In contrast, the expression of other pro-inflammatory miRNAs (miR-21, miR-146a, and miR-223) remained unchanged, suggesting a selective and regulated pattern of non-secretion. Similar phenomena have also been observed in cancer immunology. Tumor cell-derived miR-214 induces immune tolerance via Treg expansion and its inhibition reduces tumor growth (Yin et al., 2014). Tumor cells under hypoxic stress release EVs enriched with miR424, which are taken up by tumor-infiltrating dendritic and T cells. In these immune cells, miR424 suppresses the CD28–CD80/86 co-stimulatory pathway, effectively dampening T cell activation and promoting immune tolerance. Knockdown of miR424 in these EVs restores T cell function and enhances the efficacy of anti-PD-1 therapy in colorectal cancer models (Zhao et al., 2021). Thus, decreased miR-145 in adipose stem cells enhances angiogenesis (Arderiu et al., 2019), decreases miR-22 levels in cardiac fibroblast-induced pluripotent stem cells, and increases the contraction ability in a cardiomyopathy model (Kurtzwald–Josefson et al., 2020). These findings highlight a broader role for “non-secreted miRNAs” in intercellular communication and tissue regulation. Further investigation of this paradoxical phenomenon, in which the absence of specific miRNAs contributes to functional outcomes, may reveal novel principles of intercellular signaling. Importantly, isolated monocytes alone do not fully replicate the therapeutic efficacy of whole PBMC populations, likely due to the necessity of intercellular cross-talk among monocytes, Tregs, and other subtypes (Facciabene et al., 2011; Hatakeyama et al., 2019). This has been corroborated by *in vivo* studies, showing that preconditioned PBMCs, but not monocytes alone, significantly enhance neurobehavioral recovery in rodent stroke models (Hatakeyama et al., 2019).

Early-phase clinical trials have demonstrated the safety and potential efficacy of PBMC-based therapies. For example, the administration of autologous peripheral blood CD34^+^ mononuclear cells has shown promise in subacute stroke patients, with improvements in motor function and perfusion metrics (Chen et al., 2014). High purity (87%–97%) and sufficient cell dose (e.g., 3–8 × 10^6^ cells) are associated with better outcomes. Similarly, IL-4-polarized peripheral monocytes have been explored as potential therapeutic agents with immunomodulatory effects (Chernykh et al., 2016).

### Recruitment modulation

The CXCL12–CXCR4 and CCL2–CCR2 pathways are central to monocyte migration and phenotypic polarization after stroke. Pharmacological modulation of these axes presents a rational strategy for controlling inflammatory infiltration and promoting reparative responses.

One of the major mechanisms involves the CXCL12 (SDF-1)-CXCR4 axis, which plays a central role in directing PBMC migration toward ischemic brain tissue. SDF-1 expression is strongly upregulated under hypoxic conditions via HIF-1α. After cerebral ischemia, increased SDF-1 levels have been observed particularly in the peri-infarct regions, and PBMCs preferentially accumulate in these SDF-1-enriched areas, supporting the presence of a chemotactic gradient that facilitates targeted migration (Kanayama et al., 2025).

In addition to CXCL12-CXCR4 axis signaling, several adhesion molecules and chemokines have been shown to mediate PBMC trafficking and retention within ischemic brain tissue. These include VLA-4 and macrophage-1 antigen, which contribute to firm adhesion to activated endothelium. Moreover, monocyte chemoattractant protein-1/CCL2 promotes monocyte infiltration through CCR2-dependent pathways and may enhance transmigration in conjunction with integrin activation. PBMCs upregulate monocyte chemoattractant protein-1 after ischemia, further amplifying the inflammatory cascade (Hatakeyama et al., 2019). Collectively, these findings suggest that PBMC accumulation is governed by the synergistic action of hypoxia-induced chemokine signaling and endothelial adhesion molecule interactions.

CXCR4 antagonists, such as AMD3100 (plerixafor, Mozobil®), are currently used clinically in combination with granulocyte-colony stimulating factor to mobilize hematopoietic CD34^+^ stem cells into the peripheral blood for collection for autologous stem cell transplantation. The CXCR4 ligand, CXCL12, is important for hematopoietic stem cell homing to the bone marrow. AMD3100 works by antagonizing the binding of CXCL12 to its receptor CXCR4, leading to the release of hematopoietic stem cells from the bone marrow into peripheral blood (Huang et al., 2013; Walter et al., 2015; Kanayama et al., 2025). In preclinical stroke models, a study has also found that CXCR4 up-regulation, preconditioning of mesenchymal cell grafts by a small molecule and hypoxia can further enhance the homing into the stroke area (Wei et al., 2013; Li et al., 2017). However, indiscriminate CXCR4 blockade may also impair beneficial cell recruitment, necessitating targeted delivery approaches.

Engineered EVs present a novel platform for selectively targeting the CXCR4^+^ monocytes that infiltrate the brain. Translating this approach to stroke could enable the precise modulation of infiltrating monocyte subtypes (Jayasinghe et al., 2022).

Infiltrating CCR2^+^ monocytes and lymphocytes expressed Arg1, VEGF, and angiopoietin-1/2. The knockout experiments of CCR2 and administration of bone marrow-derived CCR2^+^ monocytes confirmed that CCR2+ monocytes contribute to acute post-ischemic angiogenesis and participate in functional recovery (Pedragosa et al., 2020). Infiltrating CCR2^+^ monocytes were the primary source of IL-6 in a cerebrovascular injury model and were primed to resident microglia with proliferative and proangiogenic properties (Mastorakos et al., 2021; Choi et al., 2023). Accumulating evidence indicates that angiogenesis following cerebral ischemia not only restores vascular networks but also supports neuronal survival and plasticity, thereby contributing to neurological recovery (Kanazawa et al., 2019; Hatakeyama et al., 2020a). Therefore, the administration of CCR2+ monocytes with proangiogenic properties might be reparative.

### Pharmacological and epigenetic reprogramming

Instead of cell transplantation, pharmacological interventions may reprogram endogenous PBMCs *in vivo* to adopt a neuroprotective phenotype.

#### Low-dose IL-2 therapy

Low-dose IL-2 therapy can lead to Treg expansion, and thus suppress excessive immune responses. Low-dose IL-2 is efficacious in steroid-refractory chronic graft-*versus*-host disease, with objective responses in > 50% of patients (Koreth et al., 2016). Expanding Tregs response via IL-2 may modulate post-stroke inflammation following an experimental stroke (Yuan et al., 2023).

#### Small-molecule reprogramming

Strategies aimed at converting microglia or macrophages into induced neurons in situ. Combinations of epigenetic and signaling pathway modulators (e.g., ISX9 and Forskolin) have been shown to induce neuronal phenotypes in ischemic brain tissue, offering an innovative avenue for direct *in vivo* regeneration (Ninomiya et al., 2023; Irie et al., 2023).

### Combining remote ischemic conditioning with cellular therapies

Combining cell-based therapies with interventions that enhance recovery is an emerging therapeutic strategy. Although direct clinical evidence for pairing cell therapy with rehabilitation is still limited, its potential synergistic effects justify further investigation in well-designed clinical trials (Li et al., 2020). Another promising adjunct is RIC—the application of brief, non-lethal ischemia to a limb—which has been shown to modulate systemic immune responses and promote endogenous repair mechanisms following stroke.

RIC activates HIF-1α and upregulates CXCL12, mirroring some of the pathways activated by PBMC preconditioning. Several clinical trials have evaluated the RIC. RICAMIS trial showed a modest but significant benefit in functional outcomes (Chen et al., 2022). However, RESIST trial showed that the protocol did not significantly improve functional outcomes at 90 days in patients with acute stroke (Blauenfeldt et al., 2023). Both induce cytoprotective, anti-inflammatory, and proangiogenic phenotypes via hypoxia-related signaling such as HIF-1α and CXCL12-CXCR4 axis activation (Hill et al., 2004; Kanayama et al., 2025). Notably, RIC and oxygen-glucose deprivation-PBMC therapies may have synergistic effects. RIC can prime the immune system and enhance PBMC mobilization; thus, oxygen-glucose deprivation-preconditioned PBMCs can amplify reparative signaling within the brain. Future studies should investigate whether combining RIC with cell therapy can overcome the limitations of each modality used in isolation.

## Special Considerations and Future Directions

Aging significantly affects the immune responses, which in turn influence stroke outcomes. The interaction has received increasing attention in recent years. Compared to young individuals, older adults exhibit reduced anti-inflammatory activity from the intermediate and non-classical monocytes (Wang et al., 2023; Basu et al., 2025), as well as diminished Tregs responsiveness (de la Fuente et al., 2024). These age-related changes contribute to a more pro-inflammatory state following stroke. Therefore, it is essential to consider the effects of aging on monocyte function when evaluating immune responses to stroke.

The traditional classification of monocytes into classical, intermediate, and non-classical subsets is based on surface markers such as CD14 and CD16 in humans (or Ly6C in mice). However, these phenotypes do not necessarily correlate with stable functional states. Recent single-cell and epigenomic analyses suggest that monocytes exhibit remarkable plasticity. Moreover, monocyte function is strongly determined by the epigenomic state of the progenitor cell (Rhee et al., 2023). A key challenge lies in distinguishing transient activation states after stroke from true lineage-determined functions. Specific classification of cells using a combination of surface markers, epigenetics, and function will support the cellular effects.

Prof. Savitz proposed the “bioreactor hypothesis,” which conceptually aligns with and supports the observed indirect therapeutic benefits of MNC therapies in ischemic stroke (Savitz and Cox, 2023). According to this model, peripheral organs such as the spleen and lungs serve as immediate and primary targets of intravenously administered cell-based therapies for neurological disorders. These organs function as immunological “bioreactors,” where exogenous cells modulate host immune responses, ultimately promoting neuroprotection and repair of the CNS. This concept opens the possibility of evolving toward cell-free approaches, in which the immunoregulatory effects of cell therapies are replicated through the identification and delivery of specific soluble factors, such as EVs or cytokine cocktails.

Finally, in chronic neurodegenerative diseases such as Alzheimer’s disease and amyotrophic lateral sclerosis, MDM recruitment has been associated with neuroprotective roles (Schwartz et al., 2024; Bensimon et al., 2025). Notably, a recent study in amyotrophic lateral sclerosis patients demonstrated that adding low-dose IL-2 to riluzole, a glutamate release inhibitor with potent neuroprotective effects, significantly increased the number and proportion of Tregs and reduced plasma levels of CCL2. This combination therapy led to a 48% reduction in mortality risk (hazard ratio 0.52, *P* = 0.016) and significantly slowed the rate of functional decline (Bensimon et al., 2025). These findings suggest that therapeutic strategies aimed at immunomodulation may hold promise not only for stroke but also for neurodegenerative conditions.

## Conclusion

**Figure 5:**
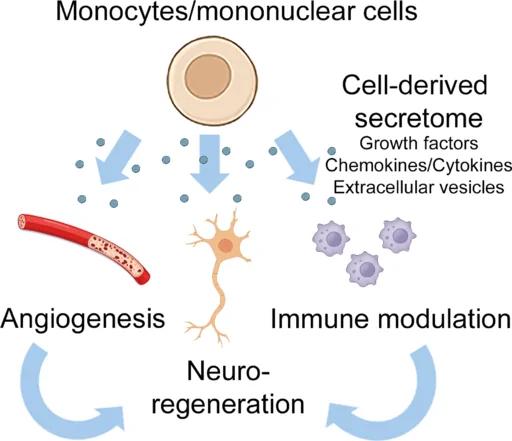
Schematic of cell-derived secretome promoting vascular and neural regeneration and immune modulation.

The crosstalk between peripheral monocytes and CNS cells is a double-edged sword in cerebrovascular diseases. While acute monocyte and PBMC infiltration exacerbates injury, later anti-inflammatory MDMs promote tissue repair through the secretome, which includes growth factors, cytokines, and EVs. This secretome and decreased levels of secretory miRNAs support angiogenesis, neuroregeneration, and immune modulation. Furthermore, angiogenesis and immune modulation have synergistic effects on neuroregeneration (**Figure 5**). Therapies targeting subset polarization, recruitment timing, or EV-mediated communication offer transformative potential. Future research must optimize the delivery methods and timing to harness the dual roles of monocytes and PBMCs in the pathophysiology and repair of cerebrovascular diseases.

## Data Availability

*Not applicable.*
